# MiR-518c-5p/miR-4524a-3p can mediate immune escape and chemotherapy resistance in triple-negative breast cancer and predict its outcome

**DOI:** 10.1186/s41065-025-00572-8

**Published:** 2025-10-21

**Authors:** Yuan Sun, Liang An, Linna Kong, Xiaoyan Liu, Huihui Zhang, Jiaqi Liu, Pengfei Qian

**Affiliations:** 1 Department of Oncology, Qujing Central Hospital of Yunnan Province (Qujing First People’s Hospital), Yunnan, 655000 China; 2 Department of Oncology Surgery, Shanghai Mengchao Cancer Hospital, Shanghai, 200000 China; 3 Department of Breast and Thyroid Surgery, Zibo Central Hospital, Zibo, 255020 China; 4 Department of Breast Surgery, Huizhou Third People’s Hospital, Affiliated Hospital of Guangzhou Medical University, No.1 Qiaodong Xuebei Street, Huicheng District, Huizhou, 516001 China

**Keywords:** MiR-518c-5p/miR-4524a-3p, Triple-negative breast cancer, Immune escape, Chemotherapy resistance

## Abstract

**Background:**

Triple-negative breast cancer (TNBC), is characterized by its highly aggressive nature, with chemotherapy resistance and immune evasion contributing to poor outcomes. The role of major histocompatibility complex class I (MHCI) downregulation in TNBC progression remains incompletely understood.

**Aim:**

The present research focused on elucidating the roles of miR-518c-5p/miR-4524a-3p in immune evasion and chemoresistance in TNBC, and evaluating their clinical significance.

**Methods:**

Bioinformatics analysis predicted miRNAs targeting *HLA-A/B/C*, validated by dual-luciferase assays. Functional studies in TNBC cell lines (MDA-MB-231/468 and ADR-resistant sublines) included chromium release, proliferation, invasion, and apoptosis assays. Clinical relevance was assessed in 88 TNBC patients and 88 controls using RT-PCR and survival analysis.

**Results:**

MiR-518c-5p/miR-4524a-3p directly targeted *HLA-A*, *HLA-B*, and *HLA-C*, downregulating MHCI expression and promoting immune evasion. Overexpression of miR-518c-5p/miR-4524a-3p enhanced TNBC cell proliferation, invasion, and chemoresistance to doxorubicin, while their inhibition reversed these effects. High expression of miR-518c-5p/miR-4524a-3p correlated with adverse clinical outcomes in TNBC patients, including shorter recurrence-free survival.

**Conclusions:**

MiR-518c-5p/miR-4524a-3p contribute to TNBC progression by facilitating immune evasion and chemoresistance. Targeting miR-518c-5p/miR-4524a-3p may represent a promising therapeutic approach for improving TNBC treatment outcomes.

**Supplementary Information:**

The online version contains supplementary material available at 10.1186/s41065-025-00572-8.

## Introduction

Breast cancer (BC) is the most common malignancy among women and ranks as one of the top three cancers globally, with continuously rising incidence and mortality rates [1]. As a highly heterogeneous disease, BC comprises distinct molecular subtypes, among which triple-negative breast cancer (TNBC)—defined by the absence of estrogen receptor (ER), progesterone receptor (PR), and human epidermal growth factor receptor 2 (HER2) expression—accounts for 15-25% of BC cases [2, 3]. The absence of therapeutic targets limits treatment options for TNBC, a disease characterized by rapid progression and dismal survival rates. Currently, chemotherapy remains the primary treatment modality for TNBC, in addition to surgical resection [4]. Doxorubicin (ADR), an anthracycline antibiotic, exhibits broad antitumor activity and can reduce the invasiveness and metastatic potential of TNBC cells by inhibiting their growth [5]. However, due to the unique biological characteristics of TNBC, patients often develop multidrug resistance to ADR during chemotherapy. This not only compromises the therapeutic efficacy of the drug but also results in a 5-year survival probability of less than 30% and significantly diminishes patients’ quality of life [6].

The high immunogenicity of TNBC and the significant presence of tumor-infiltrating lymphocytes (TILs) reflect its distinct immune microenvironment, which shows a significant correlation with the dynamic balance of tumor immune escape mechanisms [7]. Tumors can evade immune surveillance through intrinsic and extrinsic mechanisms. Intrinsic pathways involve downregulation of major histocompatibility complex class I (MHCI) surface expression, rendering cancer cells “invisible” to cytotoxic T lymphocytes (CTLs), as well as upregulation of PD-L1 suppressing T cells through PD-1 binding [8]. Extrinsic mechanisms include the downregulation of costimulatory molecules on antigen-presenting cells, secretion of inhibitory cytokines, and recruitment of regulatory T cells and myeloid-derived suppressor cells [9].

In tumor immunity, MHCI is the key mediator of adaptive immune responses, activating CD8⁺ cytotoxic T cells to precisely target and eliminate cancer cells [10, 11]. In humans, classical MHCI (*HLA-A*, *HLA-B*, and *HLA-C*) form heterodimers with β_2_-microglobulin (β_2_m) to assemble peptide-loaded complexes for immune recognition [12, 13]. It has been reported that many common cancers lose MHCI expression to varying degrees, allowing cancer cells to effectively escape CD8⁺ T cell-mediated immune surveillance [14]. Notably, dysregulation of MHCI antigen processing contributes to both primary and acquired resistance to immunotherapy, while restoring MHCI expression enhances T cell-mediated tumor clearance [10].

MicroRNAs (miRNAs) are a class of endogenous noncoding RNA molecules, typically consisting of 18-25 nucleotide units, which regulate gene expression at the post-transcriptional level, thereby influencing a variety of biological processes [15]. In the tumor microenvironment (TME), aberrant miRNA expression modulates tumor-immune crosstalk by not only controlling cancer cell proliferation and apoptosis but also subverting immune cell function to facilitate immune escape [16]. For instance, miR-375 downregulation in gastric cancer promotes inflammatory cytokine release and impairs immune cell differentiation [17], while miR-148/152 suppress *HLA-G* expression to disrupt antigen presentation [18]. Similarly, miR-424 attenuates chemoresistance in ovarian cancer by inhibiting CD80/CTLA-4 and PD-L1/PD-1 pathways [19].

Although chemotherapy is the mainstay of TNBC treatment, the limitations of targeted therapies have prompted researchers to explore new avenues. Lately, immunotherapy has provided new treatment options for some TNBC patients and has shown promise in improving therapeutic outcomes [20–22]. The present study explores the influence of miR-518c-5p/miR-4524a-3p on TNBC cells. Utilizing bioinformatics analysis and experimental validation, we explored the roles of miR-518c-5p/miR-4524a-3p in TNBC cell immune evasion and chemoresistance and predicted their prognostic value, which may help identify potential therapeutic targets for TNBC treatment.

## Materials and methods

### Bioinformatics analysis

Potential miRNAs targeting all three MHCI loci (*HLA-A*, *HLA-B*, and *HLA-C*) were predicted using the miRDB database (http://mirdb.org/), with miR-518c-5p/miR-4524a-3p identified as common regulators. Binding sites were further predicted via the StarBase database (https://starbase.sysu.edu.cn/).

### Study population

This study enrolled 88 TNBC patients who underwent mastectomy or breast-conserving surgery at Zibo Central Hospital and 88 healthy controls. Serum was collected from consenting participants under institutional review board-approved protocols. The study protocol was approved by the Institutional Review Board of Zibo Central Hospital.

### Blood sample collection and processing

Serum samples were obtained from all participants following a minimum 12-hour overnight fast. Venous blood was collected using silica clot activator tubes (BD Vacutainer, 351060) and incubated at room temperature for 30 min to facilitate clot formation. The samples were then subjected to centrifugation at 3000 × g for 15 min under 4 °C conditions to isolate serum. The resulting supernatant was meticulously transferred into sterile RNase-free tubes, ensuring avoidance of the pellet. Samples were preserved at -80 °C pending subsequent RNA extraction. Total RNA isolation was conducted for all specimens within one month following collection.

### Cell culture and transfection

The TNBC parental cell lines MDA-MB-231/468 were purchased from Procell (CL-0150 and CL-0290), and the ADR-resistant cell lines MDA-MB-231/ADR and MDA-MB-468/ADR were obtained from Warner Bio (NYZQ0018, and SB-HU4935). And all TNBC cell lines were cultured in high-glucose DMEM containing 10% fetal bovine serum (FBS). MDA-MB-231/ADR and MDA-MB-468/ADR were cultured in high-glucose DMEM medium supplemented with 1.0 µg/mL ADR (Macklin, D937611) to maintain their drug-resistant phenotypes. The maintenance concentration was determined according to the supplier’s protocol. The human normal breast epithelial cell line MCF-10 A was obtained from ATCC (CRL-10317) and cultured in DMEM medium with 10% FBS. CD8⁺ T cells were obtained from ATCC (PCS-800-017).

The overexpression of MHCI (oeMHCI), empty vector plasmids and lentivirus-mediated knockdown (shMHCI) were constructed by ThermoFisher (USA). Mimics and inhibitors of miR-518c-5p and miR-4524a-3p were synthesized by RiboBio (China). For transfection, cells were seeded in 6-well plates at a density of 2 × 10⁵ cells per well and cultured overnight. Then, transfection was performed using Lipofectamine 3000 (ThermoFisher, L3000015) according to the manufacturer’s instructions, with the following specifications: miRNA mimics and inhibitors were used at final concentrations of 50 nM and 100 nM, respectively, while 2.0 µg per well of each plasmid (oeMHCI, empty vector, shMHCI, or non-targeting shRNA control) was transfected. Corresponding negative controls were included in parallel in all experiments. After 48 h of transfection, the culture medium was removed, and the cells were passaged 3-4 times prior to subsequent experiments.

### Chromium release assay

MDA-MB-231 and MDA-MB-468 cells were transfected and labeled with ⁵¹Cr for 1 h at 37 °C. Subsequently, T cells (obtained from ATCC) were co-cultured with TNBC cells at various effector-to-target (E: T) ratios (40:1, 20:1, 10:1, 5:1) in 96-well V-bottom plates. For these experiments, 1 × 10⁵ TNBC cells were co-cultured with 4 × 10⁶, 2 × 10⁶, 1 × 10⁶, and 5 × 10⁵ T cells for the 40:1, 20:1, 10:1, and 5:1 E: T ratios, respectively. After 6 h, the release of ⁵¹Cr in the supernatant was measured using a Triathler Gamma counter (Finland, Hidex, 450  -0010). Statistical analysis was performed using GraphPad Prism software (version 10.1.2).

### Dual-luciferase reporter assay

MDA-MB-231 and MDA-MB-468 cells were seeded in 24-well plates at a density of 8 × 10⁴ cells per well and cultured overnight to reach approximately 70-80% confluence. Wild-type and mutant *HLA-A/B/C* reporter plasmids were constructed by GenePharma (China). The sequences of the wild-type and mutant miRNA binding sites are presented in Supplementary Table 1.

For each well, a total of 1.0 µg of DNA was used for co-transfection, consisting of 0.5 µg of reporter plasmid and 0.5 µg of either the miR-518c-5p mimic (50 nM final concentration), miR-4524a-3p mimic (50 nM final concentration), or corresponding non-targeting miRNA control (50 nM final concentration), using Lipofectamine 3000 as per the manufacturer’s protocol.

After 48 h, relative luciferase activity was quantified by employing the Dual-Luciferase Reporter Assay Kit (KeyGEN, KGE3308). Firefly and Renilla luciferase signals were quantified in sequence on a LUMIstar^®^ Omega (Germany, BMG LABTECH). Relative luminescence values were derived by dividing Firefly luciferase readings by the corresponding Renilla luciferase values for normalization.

### Western blotting

Proteins were isolated with RIPA lysis buffer (Beyotime, P0013B) containing protease inhibitors, and concentrations were determined using a BCA assay (Beyotime, P0010S). Samples (30 µg protein per lane) were separated by 10% SDS-PAGE and transferred to PVDF membranes. After blocking with 5% non-fat milk, membranes were incubated overnight at 4 °C with primary antibodies against MHCI (1:1000; univ, sc-32235) and GAPDH (1:2000; Thermo Fisher, A-2301), followed by incubation with HRP-conjugated goat anti-rabbit IgG (1:5000; Santa Cruz, sc-2004). Blots were developed with ECL reagent and captured using a ChemiDoc XRS + system (Bio-Rad). Band intensity was quantified with ImageJ software, normalized to GAPDH.

### RT-PCR

Cells were harvested, and total RNA (including miRNA) was isolated using TRIzol™ Reagent (ThermoFisher, 15596026), while miRNA from cell samples was specifically purified using the miRNeasy Mini Kit (ThermoFisher, 217004). For serum samples, miRNA was extracted with the miRNeasy Serum/Plasma Kit (Qiagen, 217184). cDNA was synthesized from 10 ng of total miRNA or total RNA using the MicroRNA Reverse Transcription Kit (ThermoFisher, 4366597) for miRNA and the PrimeScript™ RT Master Mix (TaKaRa, RR036A) for mRNA, respectively. Quantitative PCR analysis was carried out on an Applied Biosystems QuantStudio 6 Flex system. For each reaction, 1 µg of total RNA was used as template. MiRNA expression levels were detected using the TaqMan Universal Master Mix II (ThermoFisher, 4440040), while mRNA levels were assessed with the BeyoFast Probe qPCR Mix (Beyotime, D7271). The internal reference genes U6 snRNA (for cellular miRNA), GAPDH (for cellular mRNA), and cel-miR-39-3p (for serum samples) were used for normalization. All primer sequences (see Supplementary Table 2) were synthesized by Sangon Biotech (Shanghai, China). Data were analyzed using the 2^–ΔΔCt^ method.

### Cell proliferation and IC_50_ determination

Cells (5 × 10^3^) were seeded in 96-well plates, and treated with ADR or left untreated. To evaluate of ADR resistance in cell proliferation, cells were treated with ADR at concentrations of 2 and 5 µg/mL for 48 h. For IC₅₀ determination (using ADR as the test drug), cells were treated with a series of ADR concentrations (0, 0.5, 1, 1.5, 2, 2.5 µg/mL or 0, 4, 8, 16, 24, 32 µg/mL) for 48 h. After that, cell viability was assessed using 10 µL CCK-8 assay (Topscience, TS-1002). After incubation at 37 °C for 4 h, the absorbance at 450 nm was measured using a SpectraMax Paradigm (Molecular Devices, USA).

ADR was initially dissolved in DMSO (MCE, HY-Y0320) to a concentration of 32 µg/mL, followed by dilution to the experimental concentrations using high-glucose DMEM. The vehicle control group (0 µg/mL) received an equivalent volume of DMSO solution. Cell viability was calculated with blank correction, and IC₅₀ was determined by fitting the data using GraphPad Prism software.

### Transwell invasion assay

MDA-MB-231 and MDA-MB-468 cells (1 × 10^4^ per well) were placed in the upper chamber of Transwell inserts coated with Matrigel (Corning, 356234) with 5 µg/mL ADR or left untreated for invasion assays. The lower chamber was filled with the medium containing 10% FBS, and cells were incubated for 48 h before counting. The translocated cells were fixed with 4% paraformaldehyde (MCE, 768600), stained with 0.1% crystal violet (MCE, W110794), and subsequently observed and manually counted under an Olympus IX73 microscope. For each Transwell insert, at least five randomly selected fields were captured, and cell counting was performed manually.

### Cell apoptosis assay

MDA-MB-231/ADR and MDA-MB-468/ADR cells transfected with miRNA inhibitors were plated in 6-well plates at a density of 2 × 10⁵ cells per well. After overnight cell seeding, the drug was administered at a concentration of 5 µg/mL ADR for 48 h. Apoptosis was detected using an apoptosis detection kit (KeyGEN, KGA108). Briefly, all cells were harvested, washed in cold PBS, and resuspended in binding buffer before staining with Annexin V-FITC and PI for 25 min at room temperature in the dark. Samples were analyzed by flow cytometry (CytoFLEX, Beckman, USA), acquiring at least 6,000 events per sample. Gating was performed based on FSC/SSC to exclude debris, followed by analysis of Annexin V+/PI− (early apoptotic), Annexin V+/PI+ (late apoptotic), and Annexin V−/PI+ (necrotic) populations using CytExpert software.

### Statistical analysis

Quantitative data are expressed as mean ± SD (*n* ≥ 3 biological replicates). Statistical significance was determined by either a two-tailed Student’s t-test (two-group comparison) or two-way ANOVA with Tukey’s multiple comparisons test (multi-group comparison) in SPSS software (v23.0). Kaplan-Meier (K-M) analysis was used for survival analysis, and Cox regression analysis was performed to identify independent prognostic factors for disease-free survival (DFS). GraphPad Prism version 10.1.2 was used for figure generation. Statistical significance was defined as *P* < 0.05.

## Results

### Deficiency of MHCI promotes immune evasion in TNBC cells

**Fig. 1:**
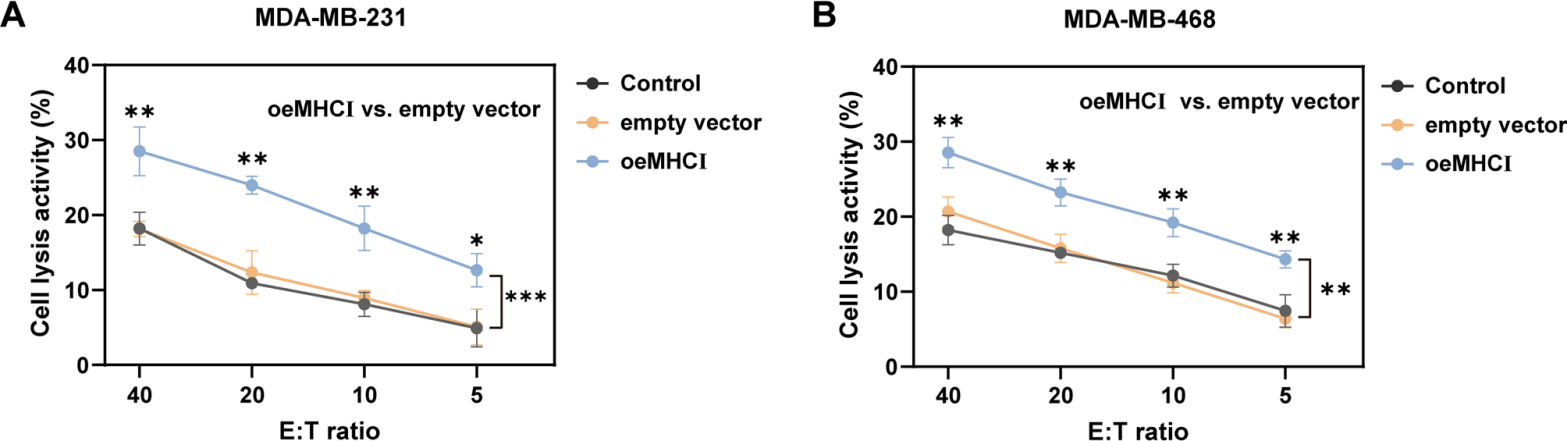
Overexpression of MHCI enhances CD8⁺ T cell-mediated cytotoxicity in TNBC cells **A-B**. ⁵¹Cr-release assays demonstrating that overexpression of MHCI in (**A**) MDA-MB-231 and (**B**) MDA-MB-468 cells significantly enhances their susceptibility to lysis by CD8⁺ T cells across multiple effector-to-target (E: T) ratios compared to empty vector controls. (*n* = 3; Student’s t-test; *p < 0.05, **p < 0.01, ***p < 0.001. Data are presented as mean ± SD.)

The chromium release assay, in which effector and target cells were co-incubated for 6 h, was employed to explore the impact of MHCI on TNBC cell lines (MDA-MB-231/468), in which MHCI overexpression was successfully achieved via plasmid transfection (Figure S1). In the Control and empty vector groups, the low lysis rate of MDA-MB-231 cells indicated reduced cytotoxic activity of CD8⁺ T cells (Fig. 1A). In contrast, MDA-MB-231 cells overexpressing MHCI significantly enhanced the cytotoxicity of CD8⁺ T cells, with lysis rates markedly higher than those of the control group across all effector-to-target (E: T) ratios (Fig. 1A), and the cytotoxic activity of CD8⁺ T cells gradually increased with higher E: T ratios (40:1 > 20:1 > 10:1 > 5:1). A similar trend was observed in MDA-MB-468 cells (Fig. 1B), suggesting that MHCI deficiency promotes immune evasion in TNBC cells.

### MiR-518c-5p/miR-4524a-3p directly suppress MHCI expression

Potential miRNAs regulating the three MHCI-encoding genes (*HLA-A*, *HLA-B*, and *HLA-C*) were predicted using the miRDB database. A Venn diagram revealed two overlapping miRNAs, miR-518c-5p/miR-4524a-3p, which were predicted to target the 3’-untranslated regions (3’-UTRs) of all three genes (Figure S2). These miRNAs exhibited potential binding sites with *HLA-A*, *HLA-B*, and *HLA-C*, indicating that they may directly target MHCI-encoding genes.

**Fig. 2:**
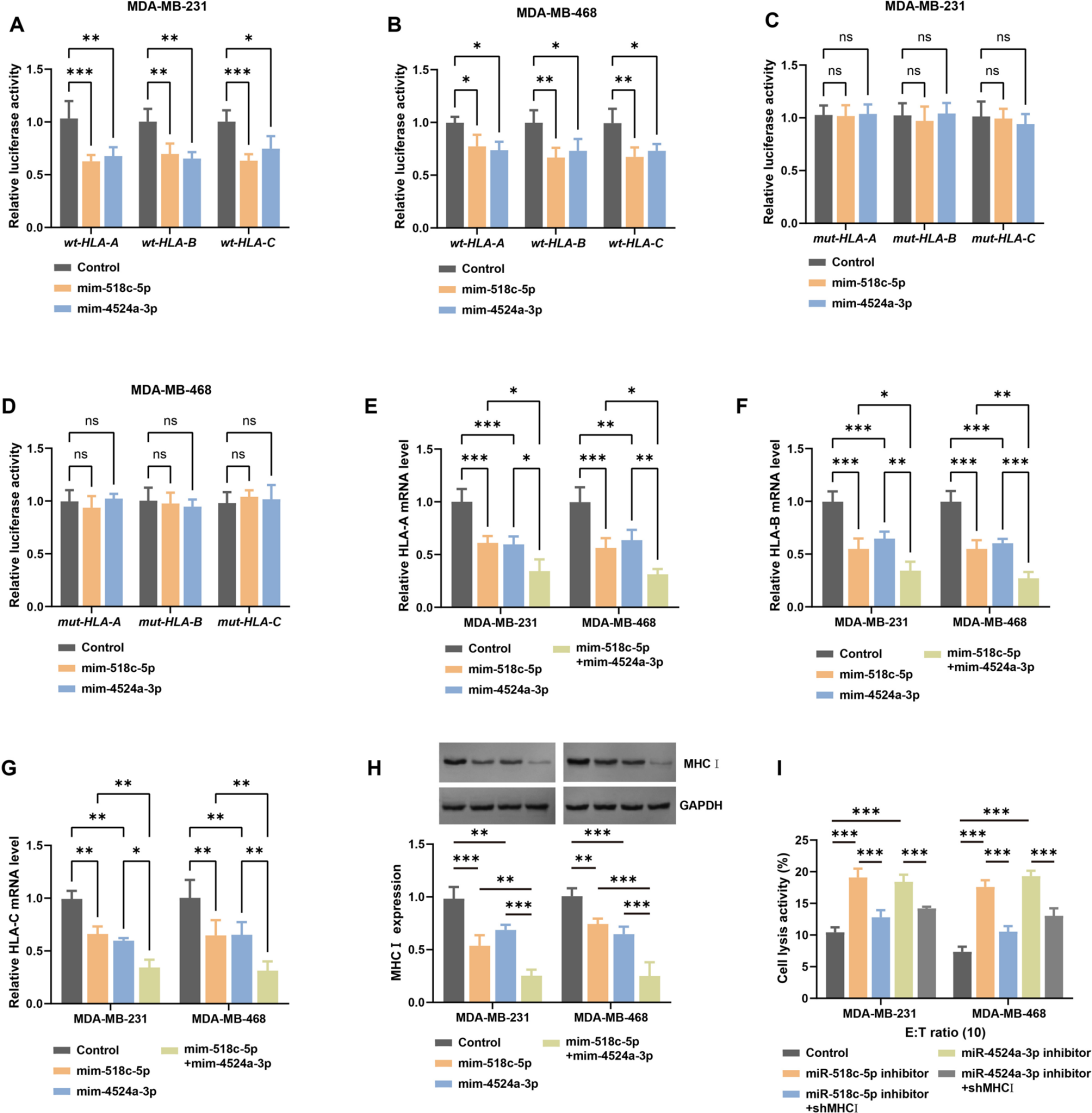
MiR-518c-5p/miR-4524a-3p negatively regulate MHCI **A-D**. Luciferase reporter assay in (**A, C**) MDA-MB-231 and (**B, D**) MDA-MB-468 cells co-transfected with miR-518c-5p mimic, miR-4524a-3p mimic, or control and a reporter plasmid containing the (**A, B**) wild-type (wt) or (**C, D**) mutant (mut) 3’-UTR of *HLA-A*, *HLA-B*, and *HLA-C*. **E-G**. RT-PCR validation of (**E**) HLA-A, (**F**) HLA-B, and (**G**) HLA-C expression after transfection with miRNA mimics. H. MHCI protein expression was analyzed by Western blotting after transfection with the indicated miRNA mimics I. Specific lysis of TNBC cells by CD8⁺ T cells at an E: T ratio of 10:1 after transfection with miRNA inhibitors with or without MHCI knockdown (shMHCI). (*n* = 3; two-way ANOVA; *p < 0.05, **p < 0.01, ***p < 0.001, ns: not significant importance. Data are presented as mean ± SD.)

Wild-type and mutant 3’-UTR sequences of *HLA-A*, *HLA-B*, and *HLA-C* vectors were constructed and introduced into MDA-MB-231/468 cells. Introduction of miR-518c-5p mimic markedly decreased luciferase reporter activity in wild-type cells of both cell lines (Fig. 2A-B), while no statistically significant alterations were detected in the mutant 3’-UTR groups (Fig. 2C-D). Similar results were obtained with miR-4524a-3p mimic (Fig. 2A-D), confirming that miR-518c-5p/miR-4524a-3p can directly target the 3’-UTR of *HLA-A*, *HLA-B*, and *HLA-C*.

To further explore the effects of miR-518c-5p/miR-4524a-3p on MHCI expression, MDA-MB-231 and MDA-MB-468 cells were transfected with miR-518c-5p mimic, miR-4524a-3p mimic, or a combination of both mimics. In MDA-MB-231 cells, overexpression of either miR-518c-5p or miR-4524a-3p significantly downregulated MHCI expression, and co-overexpression of both miRNAs further enhanced this effect (Fig. 2E-H). A similar trend was detected in MDA-MB-468 cells (Fig. 2E-H). Collectively, these findings demonstrate that miR-518c-5p/miR-4524a-3p are negative regulators of MHCI.

To explore the role of miR-518c-5p/miR-4524a-3p in TNBC immune evasion, miR-518c-5p or miR-4524a-3p inhibitors were transfected into MDA-MB-231 and MDA-MB-468 cells, combined with knocked down of MHCI expression. The E: T ratio of 10:1 was chosen for analysis owing to its position within the linear detection range of cytotoxicity, avoiding saturation effects typical of higher ratios. At an E: T ratio of 10:1, the knockdown of miR-518c-5p significantly increased the lysis rate of MDA-MB-231 and MDA-MB-468 cells, indicating enhanced CD8⁺ T cell cytotoxicity (Fig. 2I). This effect was reversed by MHCI knockdown (Fig. 2I). Similarly, inhibition of miR-4524a-3p also increased cell lysis rates, which were reversed by MHCI knockdown (Fig. 2I). These findings establish miR-518c-5p/miR-4524a-3p as negative regulators of MHCI that promote immune evasion in TNBC.

### MiR-518c-5p/miR-4524a-3p promote proliferation, and invasion of TNBC cells

**Fig. 3:**
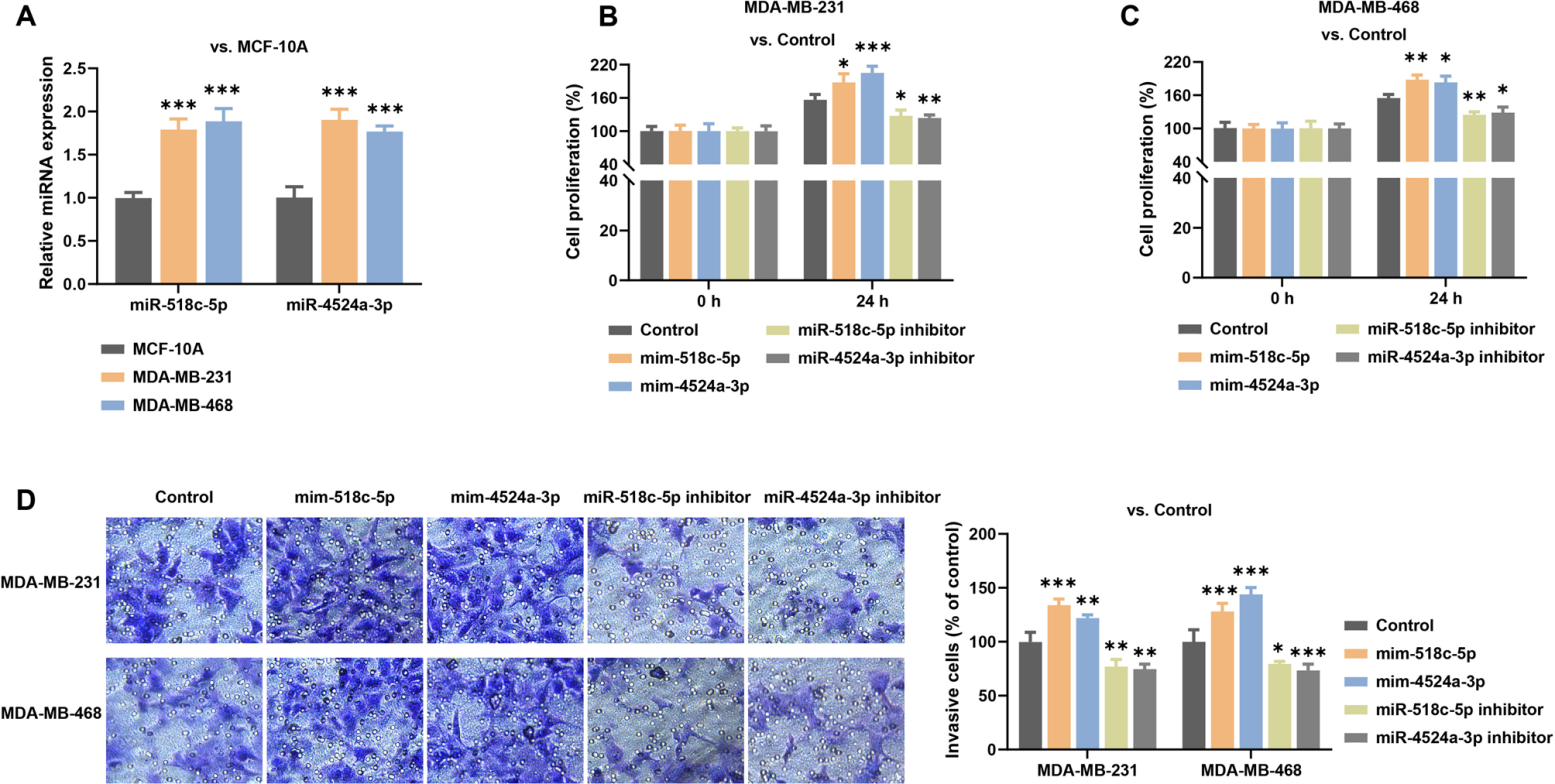
Effects of miR-518c-5p/miR-4524a-3p on TNBC cells **A**. MiR-518c-5p/miR-4524a-3p expression in MCF-10 A and TNBC cells was determined by RT-PCR. **B-C**. Cell proliferation was assessed by CCK-8 assay in (**B**) MDA-MB-231 and (**C**) MDA-MB-468 cells transfected with miRNA mimics or inhibitors. **D**. Cell invasion was evaluated by Matrigel Transwell assay. (*n* = 3; two-way ANOVA; *p < 0.05, **p < 0.01, ***p < 0.001. Data are presented as mean ± SD.)

Compared to MCF-10 A, the expression levels of miR-518c-5p/miR-4524a-3p were significantly upregulated in MDA-MB-231/468 cells (Fig. 3A). To further investigate the role of miR-518c-5p/miR-4524a-3p in TNBC cells, MDA-MB-231/468 cells were transfected with miR-518c-5p mimic, miR-4524a-3p mimic, miR-518c-5p inhibitor, or miR-4524a-3p inhibitor. Cell proliferation assays showed that, compared to the control group, overexpression of miR-518c-5p significantly promoted proliferation of MDA-MB-231 cells at 48 h post-transfection, while knockdown of miR-518c-5p suppressed growth (Fig. 3B). Similarly, overexpression of miR-4524a-3p promoted the proliferation of MDA-MB-231 cells, whereas its inhibition suppressed growth (Fig. 3B). These effects were also observed in MDA-MB-468 cells (Fig. 3C), indicating that miR-518c-5p/miR-4524a-3p promote TNBC cell proliferation.

Invasion assays in MDA-MB-231/468 cells revealed that up-regulation of miR-518c-5p or miR-4524a-3p markedly enhanced cellular invasiveness while their inhibition reduced invasiveness (Fig. 3D). Overall, miR-518c-5p/miR-4524a-3p promote both proliferation and invasion of TNBC cells.

### MiR-518c-5p/miR-4524a-3p confer chemoresistance via MDR1 upregulation

**Fig. 4:**
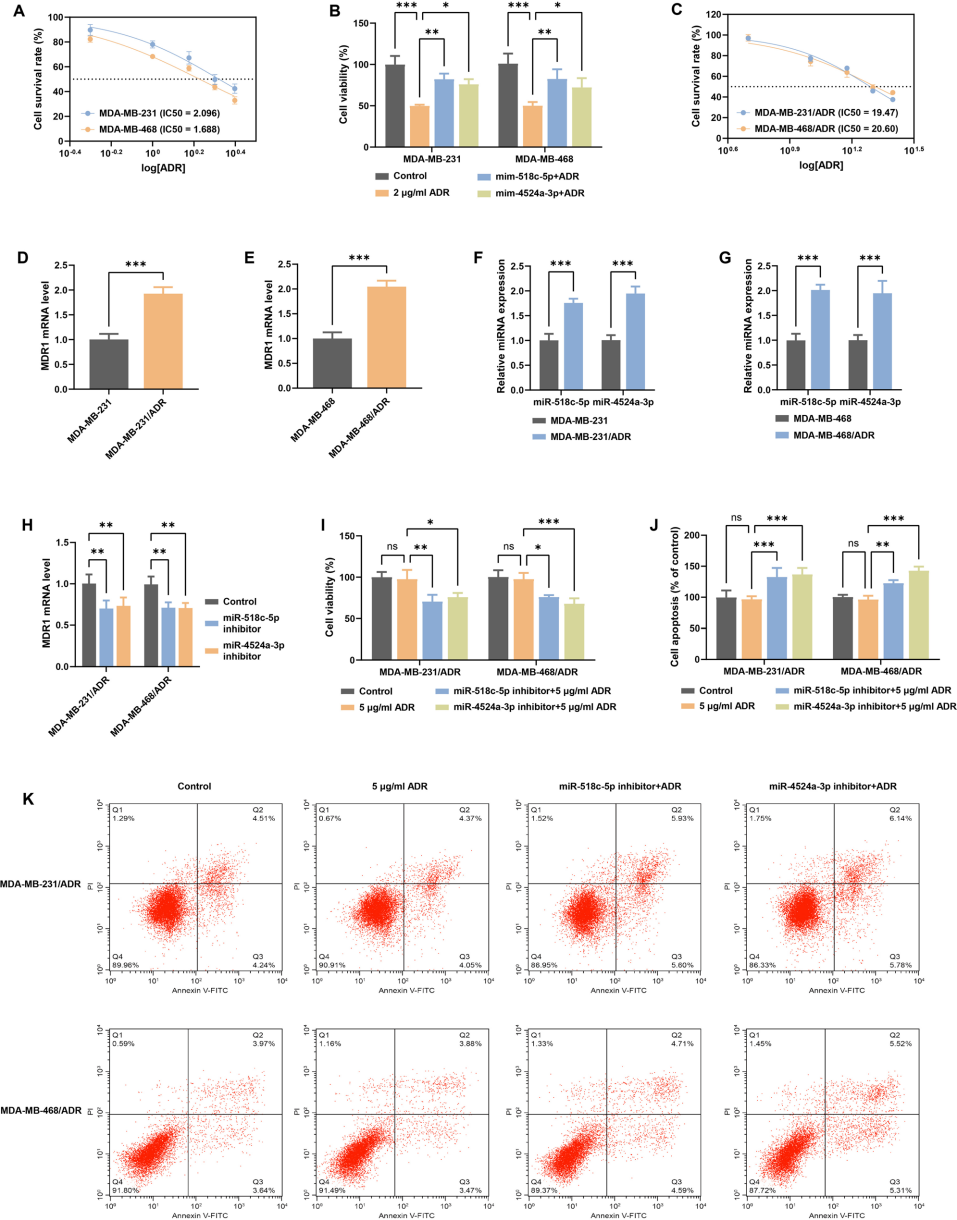
Inhibition of miR-518c-5p and miR-4524a-3p reverses chemoresistance in TNBC **A, C**. The half-maximal inhibitory concentration (IC₅₀) of Doxorubicin (ADR) was determined in (**A**) parental and (**C**) ADR-resistant TNBC cells. **B**. Cell viability of parental cells transfected with miRNA mimics and treated with ADR (2 µg/mL) for 48 h. **D-E**. Relative MDR1 mRNA expression levels in (**D**) MDA-MB-231/ADR and (**E**) MDA-MB-468/ADR cells compared to their parental cells. **F-G**. Expression of miR-518c-5p and miR-4524a-3p in parental and ADR-resistant cells: (**F**) MDA-MB-231 and MDA-MB-231/ADR, (**G**) MDA-MB-468 and MDA-MB-468/ADR. **H**. MDR1 mRNA expression in ADR-resistant cells after miRNA inhibition. **I**. Cell viability of ADR-resistant cells following transfection with miRNA inhibitors and treatment with ADR (5 µg/mL) for 48 h, measured by CCK8 assay. **J-K**. Apoptosis was measured by flow cytometry: (**J**) shows the quantitative analysis of apoptosis rates, and (**K**) shows representative flow cytometry plots corresponding to the data in (**J**). (*n* = 3; Student’s t-test and two-way ANOVA; *p < 0.05, **p < 0.01, ***p < 0.001, ns: not significant importance. Data are presented as mean ± SD.)

The IC₅₀ values of ADR in MDA-MB-231/468 cells were 2.096 and 1.688 µg/mL, respectively (Fig. 4A), providing a baseline for subsequent sensitivity assays. A series of experiments were conducted to investigate the role of miR-518c-5p/miR-4524a-3p in chemoresistance. MDA-MB-231 and MDA-MB-468 cells were transfected with miR-518c-5p or miR-4524a-3p mimics and treated with 2 µg/mL ADR (a concentration close to the IC₅₀) for 48 h. Cell viability assays showed that overexpression of miR-518c-5p/miR-4524a-3p significantly increased cell viability from approximately 50-80% compared to ADR treatment alone (Fig. 4B), indicating that miR-518c-5p/miR-4524a-3p rescued TNBC cells from ADR-induced cytotoxicity and may contribute to chemoresistance.

Multidrug-resistant MDA-MB-231/ADR and MDA-MB-468/ADR cells were used to further explore the role of miR-518c-5p/miR-4524a-3p in resistance. The IC₅₀ values of ADR in MDA-MB-231/ADR and MDA-MB-468/ADR cells were 19.47 and 20.60 µg/mL, respectively (Fig. 4C), indicating higher resistance to ADR. The mRNA expression of the key chemoresistance gene Multidrug Resistance 1 (MDR1) was significantly upregulated in MDA-MB-231/ADR and MDA-MB-468/ADR cells compared to MDA-MB-231/468 cells (Fig. 4D-E), with levels approximately double those of the control group. Expression of miR-518c-5p/miR-4524a-3p was also significantly upregulated in these resistant cells (Fig. 4F-G). Knockdown of miR-518c-5p/miR-4524a-3p significantly inhibited MDR1 mRNA expression (Fig. 4H), suggesting that they may regulate chemoresistance.

The concentration of 5 µg/mL ADR was selected as it represents one-fourth of the IC₅₀ value for resistant cells, a level sufficient to induce chemoresistance phenotypes while preserving adequate cell viability for subsequent functional assays. Treatment of ADR-resistant sublines with 5 µg/mL ADR for 48 h had no significant differences in cell viability or apoptosis compared to controls (Fig. 4I-K). However, co-treatment with miR-518c-5p or miR-4524a-3p inhibitors and ADR significantly decreased cell viability and increased apoptosis (Fig. 4I-K). These results indicate that silencing miR-518c-5p/miR-4524a-3p can inhibit MDR1 expression and reduce chemoresistance in TNBC cells.

### Clinical significance of miR-518c-5p/miR-4524a-3p in TNBC

**Fig. 5:**
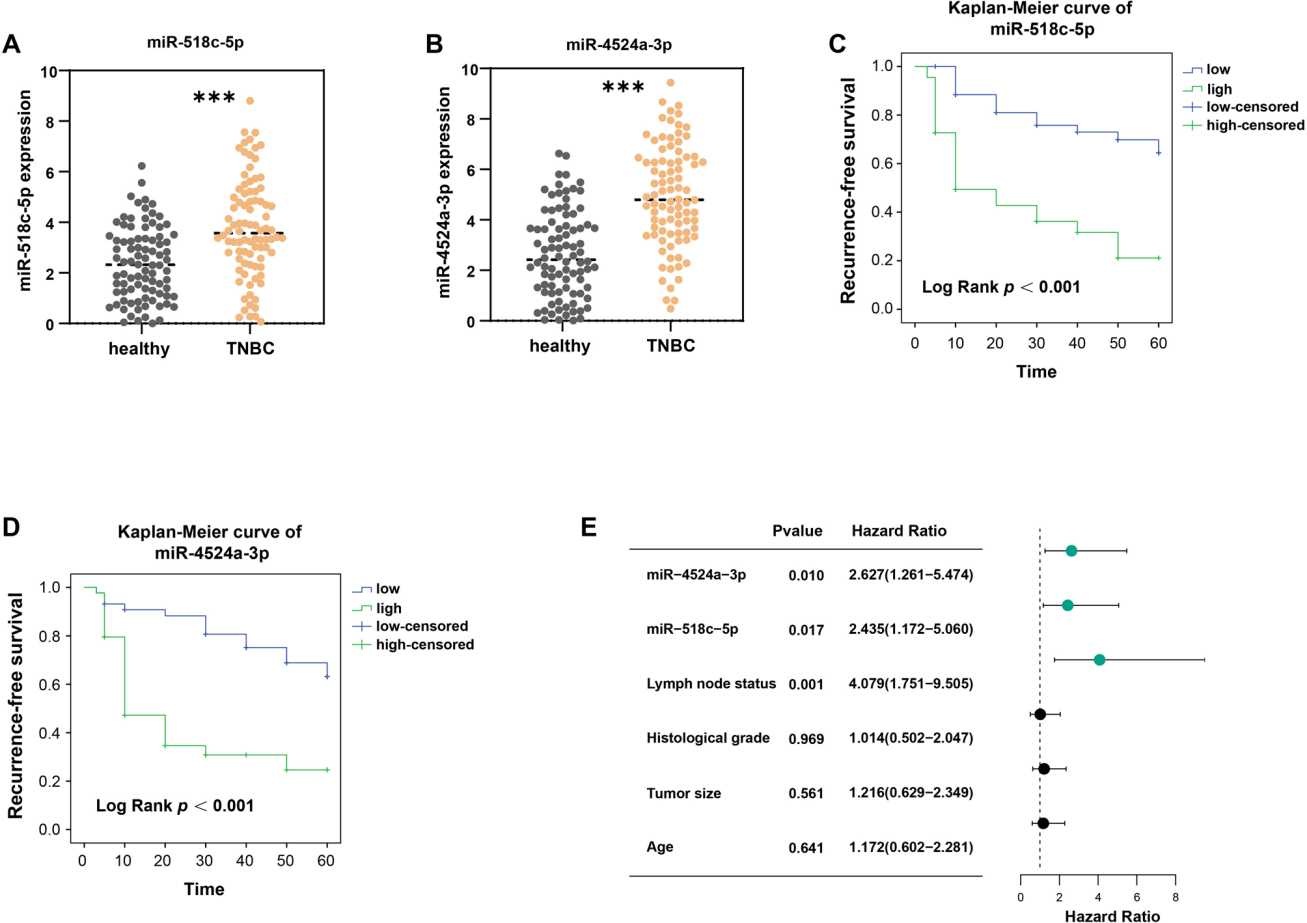
Clinical significance of serum miR-518c-5p/miR-4524a-3p in TNBC **A-B**. Scatter plots showing the expression levels of (**A**) miR-518c-5p and (**B**) miR-4524a-3p in serum samples from healthy controls (*n* = 88) and TNBC patients (*n* = 88). **C-D**. Kaplan-Meier curves for recurrence-free survival (RFS) in TNBC patients stratified by high or low expression of (**C**) miR-518c-5p and (**D**) miR-4524a-3p (log-rank test). **E**. Forest plot of multivariate Cox regression analysis for RFS. (*n* = 3; Student’s t-test; ***p < 0.001. Data are presented as mean ± SD.)

Expression profiles of miR-518c-5p/miR-4524a-3p were analyzed in serum samples from 88 TNBC patients who underwent mastectomy or breast-conserving surgery and 88 healthy controls (Fig. 5A-B). Elevated expression of miR-518c-5p/miR-4524a-3p was observed in the serum of TNBC patients. Using median expression values as the cutoff, patients were stratified into high and low miR-518c-5p/miR-4524a-3p expression cohorts. Baseline characteristics revealed significant differences in lymph node metastasis and histological grade between the high- and low-expression groups, while no significant differences were observed in tumor size or age (Tables 1 and 2). What’s more, Kaplan-Meier survival analysis showed that patients in the high-expression group had a more rapid decline in recurrence-free survival (RFS) within 5 years (Fig. 5C-D), indicating a poorer prognosis. Multivariate Cox regression revealed that TNBC recurrence was significantly associated with miR-518c-5p, miR-4524a-3p, and lymph node metastasis (Fig. 5E). Lymph node metastasis had the highest hazard ratio (HR) of 4.079, making it the strongest risk factor, while the recurrence risk associated with miR-518c-5p/miR-4524a-3p was approximately twice that of other unrelated factors.

**Table 1:** The correlation between serum miR-518c-5p and clinical characteristics

Characteristics	n (%)	Serum miR-518c-5p		p* value
		low	high	
Lymph node status				0.001
Negative	45 (51.1%)	30	15	
Positive	43 (48.9%)	14	29	
Histological grade				0.006
Ⅰ + Ⅱ	43 (48.9%)	28	15	
Ⅲ	45 (51.1%)	16	29	
Tumor size				0.088
< 2 cm	46 (52.3%)	19	27	
≥ 2 cm	42 (47.7%)	25	17	
Age				0.830
< 50	37 (42.0%)	18	19	
≥ 50	51 (58.0%)	26	25	

p*: Chi-square test

**Table 2:** The correlation between serum miR-4524a-3p and clinical characteristics

Characteristics	n (%)	Serum miR-4524a-3p		p* value
		low	high	
Lymph node status				0.006
Negative	45 (51.1%)	29	16	
Positive	43 (48.9%)	15	28	
Histological grade				0.019
Ⅰ + Ⅱ	43 (48.9%)	27	16	
Ⅲ	45 (51.1%)	17	28	
Tumor size				0.200
< 2 cm	46 (52.3%)	20	26	
≥ 2 cm	42 (47.7%)	24	18	
Age				0.280
< 50	37 (42.0%)	21	16	
≥ 50	51 (58.0%)	23	28	

p*: Chi-square test

## Discussion

Defined by negative expression of ER, PR, and HER2, TNBC does not respond to conventional endocrine or anti-HER2 therapies. Chemotherapy remains the primary treatment option for TNBC patients, however, the therapeutic efficacy is significantly compromised by immune evasion and chemoresistance [23]. Recent studies have suggested that developing combination therapies targeting immune evasion mechanisms may provide new insights into improving the prognosis of TNBC patients [15].

In this study, we employed bioinformatic analysis to predict candidate miRNAs regulating *HLA-A*, *HLA-B*, and *HLA-C* genes encoding MHCI molecules—and identified miR-518c-5p/miR-4524a-3p as dual regulators of all three loci. MHCI molecules play a central role in tumor immune responses. As critical immune recognition molecules, MHCI presents abnormal or mutated proteins from within tumor cells to the cell surface, where they can be specifically recognized by CD8⁺ cytotoxic T cells, triggering a cytotoxic response [24]. Growing evidence suggests that cancer-derived immunomodulatory miRNAs represent promising therapeutic targets [18]. Several miRNAs have been identified to regulate immune evasion in TNBC, such as miR-622 and miR-676-3p, which modulate PD-L1 ubiquitination via CMTM6 and COPS5, respectively, thereby mediating TNBC progression and immune evasion [25, 26]. Additionally, miR4435-2HG promotes immune evasion by remodeling the tumor microenvironment and serves as a potential biomarker for TNBC [27]. miR-193a-3p and miR-548c-3p have been shown to enhance immune surveillance by natural killer (NK) cells and T cell lymphocytes [28].

Our findings demonstrate that MHCI deficiency promotes immune evasion in TNBC, and miR-518c-5p/miR-4524a-3p directly suppress MHCI expression by targeting *HLA-A/B/C*, thereby impairing CD8⁺ T cell-mediated cytotoxicity. Elevated expression levels of miR-518c-5p/miR-4524a-3p were observed in TNBC cells, where they functionally enhanced proliferative capacity and invasive potential. Overexpression of miR-518c-5p/miR-4524a-3p significantly potentiated proliferative and invasive capacities in both MDA-MB-231/468 cell lines, while knockdown of these miRNAs reduced these capabilities. This is consistent with the conclusion made by Zhou et al. [29] that miR-518c-5p can promote the growth of epithelial ovarian cancer cells. Meanwhile, miR-4524a-3p can promote the progression of colorectal cancer and chronic lymphocytic leukemia [30, 31].

Adjuvant chemotherapy remains the mainstay of TNBC treatment; however, approximately 50% of patients develop chemoresistance, resulting in a short overall survival [32]. This study systematically examined the functional significance of miR-518c-5p/miR-4524a-3p in chemoresistance in TNBC cells. ADR is a first-line chemotherapeutic agent for TNBC and has demonstrated significant antitumor efficacy [33]. The study confirmed the establishment of highly resistant cell models, which recapitulate the clinical challenge of multidrug resistance.

Compared to ADR treatment alone, inhibition of miR-518c-5p/miR-4524a-3p in co-treatment with ADR treatment significantly inhibited TNBC cell viability. We further explored the resistance using multidrug-resistant MDA-MB-231/ADR and MDA-MB-468/ADR cells. These resistant cells exhibited significant chemoresistance and upregulated expression of MDR1. MDR1 encodes P-glycoprotein, which mediates drug efflux and reduces intracellular chemotherapy drug concentrations, representing a key mechanism of multidrug resistance in cancer [34]. Our findings are supported by recent studies highlighting miRNAs as pivotal regulators of TNBC chemoresistance through diverse mechanisms. For instance, in TNBC, miR-221-3p targets FSCN1 to influence gefitinib resistance, and inhibiting FSCN1 or overexpressing miR-221-3p can enhance gefitinib sensitivity, while STAT3 activation is linked to resistance and its inhibition can upregulate miR-221-3p and downregulate FSCN1 to boost gefitinib efficacy [35]. Conversely, miR-31-5p enhances sensitivity to paclitaxel (TAX) and cisplatin (DDP) by targeting PKCε and inhibiting Bcl-2, with AKT inhibition further restoring drug sensitivity [36]. We also observed significantly upregulated expression of miR-518c-5p/miR-4524a-3p in MDA-MB-231/ADR and MDA-MB-468/ADR cells. Silencing miR-518c-5p/miR-4524a-3p downregulated MDR1 expression in these resistant cells. Co-treatment with miR-518c-5p/miR-4524a-3p inhibitors and ADR significantly reduced cell viability and increased apoptosis compared to ADR treatment alone, indicating that silencing miR-518c-5p/miR-4524a-3p alleviates chemoresistance in TNBC cells and represents a potential target for overcoming chemoresistance.

While our study has established the crucial role of these miRNAs in regulating MHCI -mediated immune evasion and chemoresistance through MDR1, their potential impact on other immune regulatory mechanisms remains an open question. Although currently there are no prior studies linking miR-518c-5p or miR-4524a-3p to other immune checkpoints such as PD-L1, future investigations should explore whether these miRNAs have broader roles in modulating the tumor immune microenvironment. Examining the effect of miRNA manipulation on PD-L1 expression and other checkpoint molecules would help determine if therapeutic targeting of these miRNAs could provide dual benefits by simultaneously enhancing tumor immunogenicity and overcoming chemoresistance.

Serum miRNA detection, as a non-invasive tool, has shown great potential in the diagnosis and monitoring of diseases, especially cancer, due to its extremely high sensitivity in effectively identifying trace nucleic acids released by tumors [37]. This study used stem-loop reverse transcription qPCR to detect miR-518c-5p and miR-4524a-3p. This method can highly specifically distinguish closely related miRNA isoforms [38], thus showing excellent sensitivity and specificity in differentiating TNBC from healthy controls. The significant correlation between expression levels and clinical outcomes further supports their reliable clinical application value. We should also recognize the inherent challenges of this strategy. The expression of serum miRNAs is susceptible to other diseases, physiological states, and pre-analytical factors (such as sample processing and normalization procedures), which may limit their accuracy as specific biomarkers [39]. To improve diagnostic performance, future work needs to advance the standardization of the entire analytical process, adopt robust normalization strategies, and consider integrating multiple biomarkers into diagnostic panels [40]. The findings from this study provide valuable insights and contribute to the ongoing efforts towards developing highly specific miRNA panels, which may hold promise for future clinical applications.

Disease progression and recurrence in TNBC patients typically occur within the first 3-5 years after diagnosis [41]. Therefore, we further explored the relationship between miR-518c-5p/miR-4524a-3p expression and TNBC prognosis. Our analysis of 88 TNBC patients revealed elevated serum levels of miR-518c-5p/miR-4524a-3p compared to healthy controls, with high expression correlating with lymph node metastasis and advanced histologic grade. Kaplan-Meier curves demonstrated that elevated expression of miR-518c-5p /miR-4524a-3p showed a significant correlation with shorter recurrence-free survival in TNBC patients. Cox regression analysis indicated that miR-518c-5p/miR-4524a-3p were correlated with TNBC recurrence. Overall, we propose that high expression of miR-518c-5p/miR-4524a-3p may serve as a prognostic indicator in TNBC.

While this study offers novel findings, certain limitations warrant acknowledgment. Firstly, the mechanistic insights are primarily derived from in vitro studies; future investigations would be strengthened by in vivo validation using animal models-for instance, humanized mouse models-to more accurately mimic the intricate tumor microenvironment. Secondly, although a regulatory link to MDR1 was identified, the precise molecular mechanisms by which these miRNAs influence MDR1 expression merit further investigation. Lastly, the prognostic value of these serum miRNAs should be verified in larger, multi-center prospective cohorts.

## Conclusion

In conclusion, our study indicates that miR-518c-5p/miR-4524a-3p may contribute to immune evasion, proliferation, invasion, and chemoresistance in TNBC by targeting MHCI molecules and regulating MDR1 expression. The elevated expression of miR-518c-5p/miR-4524a-3p in TNBC patients correlates with adverse clinical outcomes, suggesting potential value as biomarkers. These findings provide insights into the multifaceted roles of these miRNAs in TNBC progression and highlight the need for further investigation into their therapeutic targeting potential.

## Supplementary Information

Below is the link to the electronic supplementary material.

Supplementary Material 1: Supplementary Fig. 1. Validation of MHCI transfection efficiency by Western blotting. A-B. Western blotting of MHCI protein levels in (A) MDA-MB-231 cells and (B) MDA-MB-468 cells transfected with either empty vector or oeMHCI. C-D. Western blotting of MHCI protein levels in (C) MDA-MB-231 cells and (D) MDA-MB-468 cells transfected with either shNC or shMHCI. (*n* = 3; Student's t-test; ***p < 0.001, ns: not significant importance). Data are presented as mean ± SD

Supplementary Material 2: Supplementary Table 1. Sequences of the wild-type and mutant miRNA binding sites in the 3′-UTRs of *HLA-A, HLA-B*, and *HLA-C*

Supplementary Material 3: Supplementary Table 2. RT-PCR primer sequences for the detection of miRNAs and mRNAs

Supplementary Material 4: Supplementary Fig. 2. Predicted miRNAs targeting MHCI and binding sites. A. Venn diagram of miRNAs predicted by miRDB to target *HLA-A*, *HLA-B*, and *HLA-C* (Target Score ≥ 80). B. Schematic representation of the conserved binding sites for miR-518c-5p and miR-4524a-3p within the 3’-UTRs of the *HLA-A*, *HLA-B*, and *HLA-C* genes

## Data Availability

No datasets were generated or analysed during the current study.

## Abbreviations

ADR: Doxorubicin
BC: Breast cancer
CTLs: Cytotoxic T lymphocytes
DFS: Disease-free survival
ER: Estrogen receptor
HER2: Human epidermal growth factor receptor 2
K-M: Kaplan-Meier
MDR1: Multidrug resistance protein 1
MHCI: Major histocompatibility complex class I
MiRNAs: MicroRNAs
PR: Progesterone receptor
TME: Tumor microenvironment
TILs: Tumor-infiltrating lymphocytes
TNBC: Triple-negative breast cancer
