# Supplementary figures and images for: MiR-518c-5p/miR-4524a-3p can mediate immune escape and chemotherapy resistance in triple-negative breast cancer and predict its outcome
Source: Hereditas. 2025 Oct 21;162:216. doi: 10.1186/s41065-025-00572-8 (PMC12538995; doi:10.1186/s41065-025-00572-8)

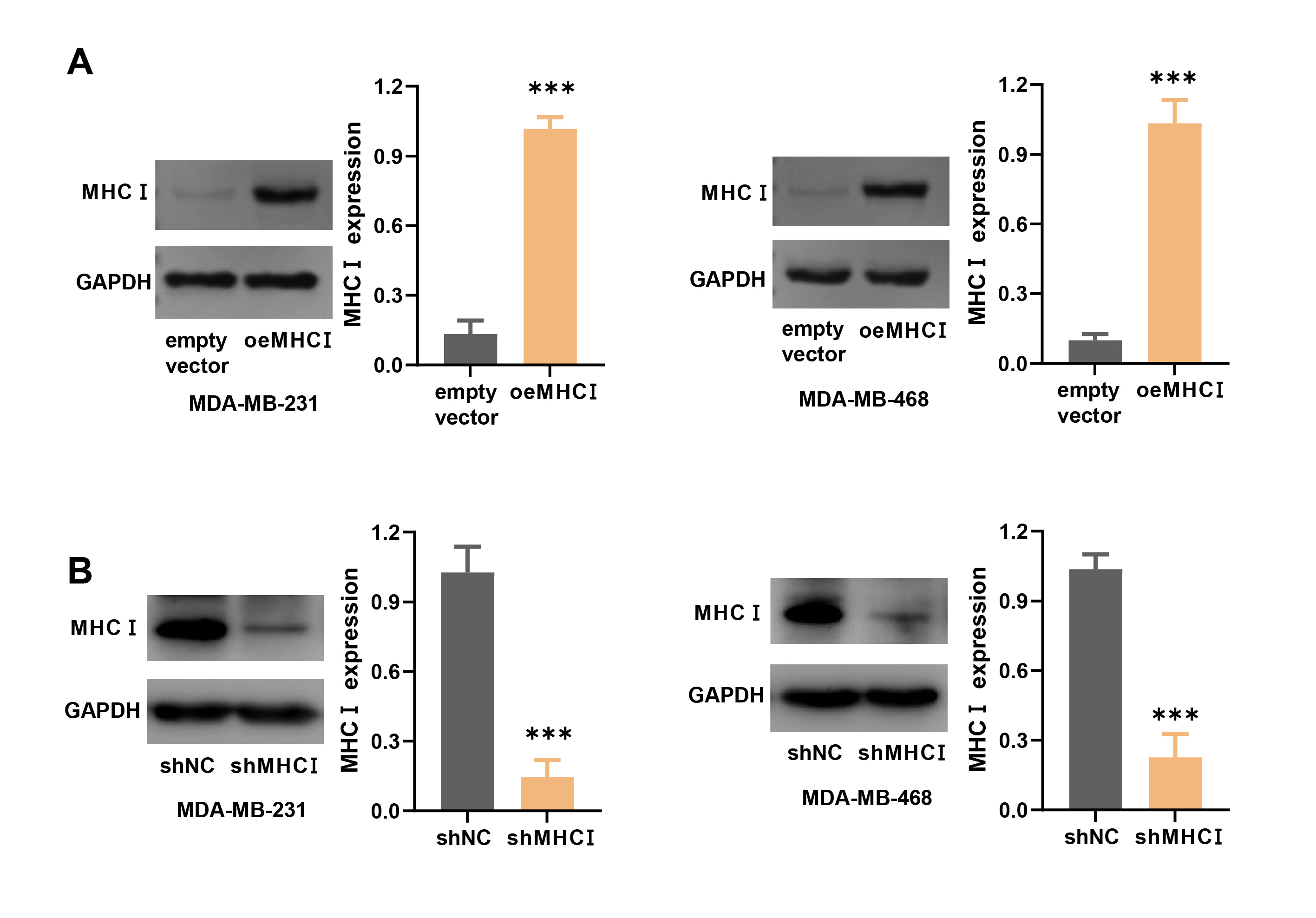

Supplement: Supplementary file 1 — Supplementary Material 1: Supplementary Fig. 1. Validation of MHCI transfection efficiency by Western blotting. A-B. Western blotting of MHCI protein levels in (A) MDA-MB-231 cells and (B) MDA-MB-468 cells transfected with either empty vector or oeMHCI. C-D. Western blotting of MHCI protein levels in (C) MDA-MB-231 cells and (D) MDA-MB-468 cells transfected with either shNC or shMHCI. (n = 3; Student's t-test; ***p < 0.001, ns: not significant importance). Data are presented as mean ± SD [file 41065_2025_572_MOESM1_ESM.jpg]

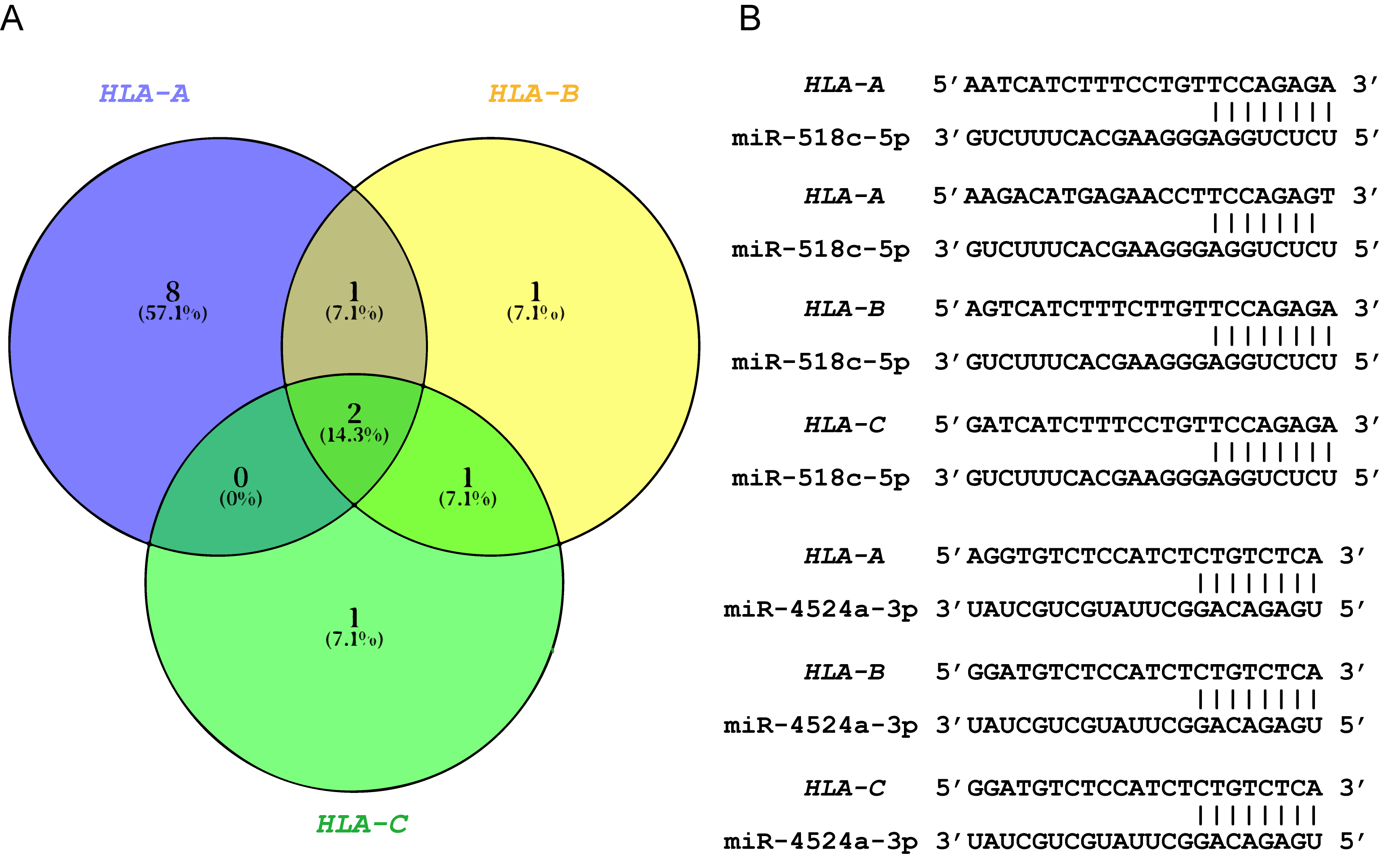

Supplement: Supplementary file 4 — Supplementary Material 4: Supplementary Fig. 2. Predicted miRNAs targeting MHCI and binding sites. A. Venn diagram of miRNAs predicted by miRDB to target HLA-A, HLA-B, and HLA-C (Target Score ≥ 80). B. Schematic representation of the conserved binding sites for miR-518c-5p and miR-4524a-3p within the 3’-UTRs of the HLA-A, HLA-B, and HLA-C genes [file 41065_2025_572_MOESM4_ESM.tif]
