# Supplementary material for: MiR-518c-5p/miR-4524a-3p can mediate immune escape and chemotherapy resistance in triple-negative breast cancer and predict its outcome
Source: Hereditas. 2025 Oct 21;162:216. doi: 10.1186/s41065-025-00572-8 (PMC12538995; doi:10.1186/s41065-025-00572-8)
Supplement: Supplementary file 2 — Supplementary Material 2: Supplementary Table 1. Sequences of the wild-type and mutant miRNA binding sites in the 3′-UTRs of HLA-A, HLA-B, and HLA-C [file 41065_2025_572_MOESM2_ESM.docx]

Supplementary Table 1. Sequences of the wild-type and mutant miRNA binding sites in the 3′-UTRs of *HLA-A*, *HLA-B*, and *HLA-C*.

| Gene | Targeting miRNA | Type | Nucleotide sequence (5’→3’) | 3’UTR position (nt) |
| --- | --- | --- | --- | --- |
| *HLA-A* | miR-518c-5p | wt#1 | AATCATCTTTCCTGT**TCCAGAGA** | 201 to 223 |
|  |  | wt#2 | AAGACATGAGAACC**TTCCAGAG** | 205 to 426 |
|  |  | mut#1 | AATCATCTTTCCTGT**CTTGAGAG** | 201 to 223 |
|  |  | mut#2 | AAGACATGAGAACC**CCTTGAGA** | 205 to 426 |
| *HLA-A* | miR-4524a-3p | wt | AGGTGTCTCCATCT**CTGTCTCA** | 234 to 255 |
|  |  | mut | AGGTGTCTCCATCT**ACACTCTG** | 234 to 255 |
| *HLA-B* | miR-518c-5p | wt | AGTCATCTTTCTTGT**TCCAGAGA** | 211 to 233 |
|  |  | mut | AGTCATCTTTCTTGT**CTTGAGAG** | 211 to 233 |
|  | miR-4524a-3p | wt | GGATGTCTCCATCT**CTGTCTCA** | 243 to 264 |
|  |  | mut | GGATGTCTCCATCT**TCACTCTG** | 243 to 264 |
| *HLA-C* | miR-518c-5p | wt | GATCATCTTTCCTGT**TCCAGAGA** | 201 to 223 |
|  |  | mut | GATCATCTTTCCTGT**CTTGAGAG** | 201 to 223 |
|  | miR-4524a-3p | wt | GGATGTCTCCATCT**CTGTCTCA** | 233 to254 |
|  |  | mut | GGATGTCTCCATCT**TCACTCTG** | 233 to254 |
