# Supplementary material for: MiR-518c-5p/miR-4524a-3p can mediate immune escape and chemotherapy resistance in triple-negative breast cancer and predict its outcome
Source: Hereditas. 2025 Oct 21;162:216. doi: 10.1186/s41065-025-00572-8 (PMC12538995; doi:10.1186/s41065-025-00572-8)
Supplement: Supplementary file 3 — Supplementary Material 3: Supplementary Table 2. RT-PCR primer sequences for the detection of miRNAs and mRNAs [file 41065_2025_572_MOESM3_ESM.docx]

Supplementary Table 2. RT-PCR primer sequences for miRNA and mRNA analysis.

| Primer name | Sequence 5’-3’ |
| --- | --- |
| miR-518c-5p-F | ATGGTTCGTGGGTCTCTGGAGGGAAGCACTTTC |
| miR-518c-5p-R | GTGCAGGGTCCGAGGT |
| miR-4524a-3p-F | TCCCTTGGCTCTGAGTGAGAAGCACTTTC |
| miR-4524a-3p-R | GTGCAGGGTCCGAGGT |
| MDR1-F | GAGAGATCCTCACCAAGCGG |
| MDR1-R | CGAGCCTGGTAGTCAATGCT |
| *HLA-A-F* | GGACTCACACAGAAACTCAGAGC |
| *HLA-A-R* | AGGGTTCCTAAAGCATTCACTCC |
| *HLA-B-F* | CCCTGGTTTCCACAGACAGATCC |
| *HLA-B-R* | CACACTGCAGCACACAATCAGG |
| *HLA-C-F* | AGCTCACTGTCTGGCATCAAGTTCC |
| *HLA-C-R* | CTCAGGCCAAGTGCTGTTTTGTGG |
| GAPDH-F | CAATGACCCCTTCATTGACC |
| GAPDH-P | TTGATTTTGGAGGGATCTCG |
| U6-F | CTCGCTTCGGCAGCACA |
| U6-R | AACGCTTCACGAATTTGCGT |
| cel-miR-39-3p | ACACTCCAGCTGGGTCACCGGGTGTAAATCAGCTTG |
| cel-miR-39-3p | GTGCAGGGTCCGAGGT |
